# Protocol for a multi-center randomized controlled trial to evaluate the benefits of exercise incentives and corticosteroid injections in osteoarthritis of the knee (MOVE-OK)

**DOI:** 10.1186/s13063-022-06529-w

**Published:** 2022-07-27

**Authors:** William Leach, Caleigh Doherty, Marianna Olave, Bryant R. England, Katherine Wysham, Gail Kerr, Mercedes Quinones, Alexis Ogdie, Dan White, Tuhina Neogi, Carla R. Scanzello, Joshua F. Baker

**Affiliations:** 1 Corporal Michael J. Crescenz VA Medical Center, Philadelphia, PA USA; 2 Perelman School of Medicine, University of Pennsylvania, 5th Floor White Building, 3400 Spruce Street, Philadelphia, PA 19104 USA; 3 Medicine Service, VA Nebraska-Western Iowa Health Care System and Department of Internal Medicine, Division of Rheumatology & Immunology, University of Nebraska Medical Center, Omaha, NE USA; 4 VA Puget Sound Health Care System and University of Washington, Seattle, WA USA; 5 Washington DC VA Medical Center, Washington, D.C USA; 6 University of Delaware, Newark, DE USA; 7 Boston University School of Medicine, Boston, MA USA; 8 Department of Biostatistics, Epidemiology, and Informatics, Perelman School of Medicine, University of Pennsylvania, Philadelphia, PA USA

**Keywords:** Osteoarthritis of the knee, Corticosteroid injections, Exercise, Behavioral incentives, Randomized controlled trial, Crossover design, Multi-center

## Abstract

**Background:**

Knee osteoarthritis (KOA) is a high-priority problem among the aging population. While exercise has been shown to be beneficial in management of the disease, scalable and low-cost interventions to improve exercise in this population are lacking. Recent controversy over the value of corticosteroid injections for palliation has also arisen. Therefore, we designed a randomized, double-blind, placebo-controlled clinical trial with a 2-period crossover design to study (1) behavioral incentives to promote exercise and (2) corticosteroid injections to reduce pain and improve function in patients with KOA when compared to lidocaine only.

**Methods:**

The study design is a pragmatic factorial and crossover randomized clinical trial. Patients with KOA who are deemed eligible by their provider to receive knee injections and are able to walk without assistive devices will be recruited from clinical practices at four sites within the Veterans Affairs (VA) Health System in the USA. In total, 220 participants will be randomized to receive social incentives with gamification (i.e., incorporation of game elements) to promote exercise and compared to controls that receive a Fitbit but no incentive. Each patient will also be assigned to receive a blinded corticosteroid injection and a lidocaine-only injection in random order. The primary outcomes are the change in average daily step counts from baseline and the change in Knee Osteoarthritis Outcome Score (KOOS) from baseline. The study team will continuously collect step count, heart rate, and sleep data using activity monitors and patient-reported outcomes using the Way to Health (WTH) platform at two four-week intervals over eight months of follow-up. Mixed effects regression incorporating all available data points will be used for analysis.

**Discussion:**

The “Marching on for Veterans with Osteoarthritis of the Knee” (MOVE-OK) trial will take a pragmatic approach to evaluate (1) whether incentives based on behaviorally enhanced gamification can improve physical activity in this patient population and (2) whether corticosteroids injections reduce pain and disability in patients with KOA. Results of this trial will help to direct clinical practice and inform management guidelines.

**Trial registration:**

ClinicalTrials.gov NCT05035810. Registered on 5 September 2021.

**Supplementary Information:**

The online version contains supplementary material available at 10.1186/s13063-022-06529-w.

## Administrative information

Note: the numbers in curly brackets in this protocol refer to SPIRIT checklist item numbers. The order of the items has been modified to group similar items (see http://www.equator-network.org/reporting-guidelines/spirit-2727-statement-defining-standard-protocol-items-for-clinical-trials/).

**Table:** 

Title {1}	Protocol for a multi-center randomized controlled trial to evaluate the benefits of exercise incentives and corticosteroid injections in patients with osteoarthritis of the knee.
Trial registration {2a and 2b}.	NCT05035810 [ClinicalTrials.gov] [registered 2021–09-05] https://clinicaltrials.gov/ct2/show/NCT05035810?term=move+ok&cond=Osteoarthritis%2C+Knee&draw=2&rank=1
Protocol version {3}	Version #2 (09/13/2021)
Funding {4}	This trial is funded by a Rehabilitation Research & Development Merit Award (I01 RX003644). Dr. Baker is also funded by a Clinical Science Research & Development Merit Award (I01 CX001703). Dr. England is supported by a Veterans Affairs Career Development Award (IK2 CX002203).
Author details {5a}	1)Corporal Michael J. Crescenz VA Medical Center, Philadelphia, PA, USA 2)Perelman School of Medicine, University of Pennsylvania, Philadelphia, PA, USA 3)Department of Biostatistics, Epidemiology, and Informatics, Perelman School of Medicine, University of Pennsylvania, Philadelphia, PA, USA 4)Medicine Service, VA Nebraska-Western Iowa Health Care System and Department of Internal Medicine, Division of Rheumatology & Immunology, University of Nebraska Medical Center, Omaha, NE, USA 5)VA Puget Sound Health Care System and University of Washington, Seattle, WA, USA 6)Washington DC VA Medical Center, Washington, D.C., USA 7)University of Delaware, Newark, DE, USA 8)Boston University School of Medicine, Boston, MA, USA
Name and contact information for the trial sponsor {5b}	VA Rehabilitation Research & Development 810 Vermont Avenue, NW Washington, DC 20,420 (USA)
Role of sponsor {5c}	The study sponsor does not play any role in study design, collection, management, analysis, or interpretation of data; writing of the report, nor the decision to submit the report for publication.

## Introduction

### Background and rationale {6a}

Knee osteoarthritis (KOA) is a high-priority problem among the aging population, and in particular, among veterans [1–3]. Notably, very few interventions have been shown to be effective. Interventions to increase physical activity that are low cost and may be useful to address many persistent symptoms (i.e., fatigue, pain, physical function) and comorbid conditions (i.e., obesity, sarcopenia, cardiovascular disease) in patients with KOA [4–9]. Recent studies have articulated both the quality and quantity of physical activity necessary to reduce the symptomatic burden of hip and knee osteoarthritis [10]. Current organizational guidelines for the non-operative management of KOA include strong recommendations for the inclusion of exercise in programs and promotion of greater physical activity in management plans [11]. Despite these recommendations, clinicians often do not offer first-line therapies such as physical therapy and lifestyle intervention for the condition [12]. While exercise is accepted as an important aspect of management, effective strategies to promote behavioral changes in this population are lacking. First-line management options including exercise often are not discussed in clinical settings, while use of non-steroidal anti-inflammatories and opioids continues to rise [12].

A number of concepts from cognitive psychology can explain the relationship between human behavior and decision-making, referenced by researchers in a field known as behavioral economics [13]. Theorists in behavioral economics seek to explain how and why people make decisions under uncertainty and to use small changes in the form of incentives to evaluate resulting changes in behavior [14, 15]. People are often motivated by the experience of past rewards and are heavily influenced by emotions such as loss and regret aversion [16, 17]. Researchers have demonstrated that behavioral incentives can be of value in numerous disease settings. These incentives, however, have not been studied in the setting of KOA.

Despite a lack of high-quality supportive data, clinicians routinely use intra-articular corticosteroids to manage KOA. A recent Cochrane review indicates that the level of evidence to support improvements in symptoms following the use of corticosteroid injections is low [18, 19]. A recent large randomized trial demonstrated no significant difference in symptom improvement among patients receiving corticosteroid injections as compared to those receiving saline injections when evaluated at 3-month intervals, as well as a slight decline in cartilage thickness on magnetic resonance imaging (MRI) among those receiving corticosteroids [20]. However, prior studies have suggested that peak benefit of corticosteroids would be expected at 4–8 weeks, and guidelines have largely acknowledged the value of short-term benefits [19]. Thus, there remains a fundamental knowledge gap as to the true efficacy of these injections and their impact on physical activity. It is paramount to clearly define the risk/benefit profile of this treatment option.

For patients with KOA to receive the best care, more clinical trials need to be conducted with the goal of informing common practice. We designed a randomized clinical trial with a factorial design (Table 1) to test the effectiveness of behaviorally enhanced gamification (i.e., incorporation game playing elements) as well as social incentives to promote exercise, and to test the effectiveness of corticosteroid injections to reduce pain and improve function in patients with KOA. Additionally, we designed the trial using a communication technology, the Way To Health (WTH) platform, to overcome the logistical and methodological difficulties that are common in behavioral economics trials. The remote components of this protocol present novel opportunities to improve upon common practice and scale the intervention across health care systems or patient populations.

**Table 1:** Overview of the study’s factorial design

	**Social incentive and gamification**	**No social incentive or gamification**
Corticosteroid (plus lidocaine) injection first	Group 1	Group 2
Placebo (lidocaine only) injection first	Group 3	Group 4

## Objectives {7}

There are two primary study objectives: (1) To determine whether an incentive based on behaviorally enhanced gamification can improve physical activity among patients with KOA and reduce self-reported pain and disability and (2) to determine if corticosteroid injections can reduce pain and disability in patients with KOA when compared to lidocaine-only injections.

## Trial design {8}

The study is a randomized trial that incorporates a factorial design and crossover intervention (Table 1). The factorial design means that participants will be randomized to one of 4 unique groups depending on whether they will receive no intervention, one intervention, or both of the interventions. Those that do not receive corticosteroids at the time of randomization will crossover at 4 months and receive corticosteroid, while those that did receive it will receive lidocaine only. Both participants and providers will be blinded to injection type [corticosteroid v. lidocaine only (placebo)]. We hypothesize that those that receive the exercise incentive will achieve higher step counts over 32 weeks. We also hypothesize that the symptoms of KOA will be reduced over 3 months among those receiving corticosteroid injections compared to those receiving lidocaine only.

**Fig. 1:**
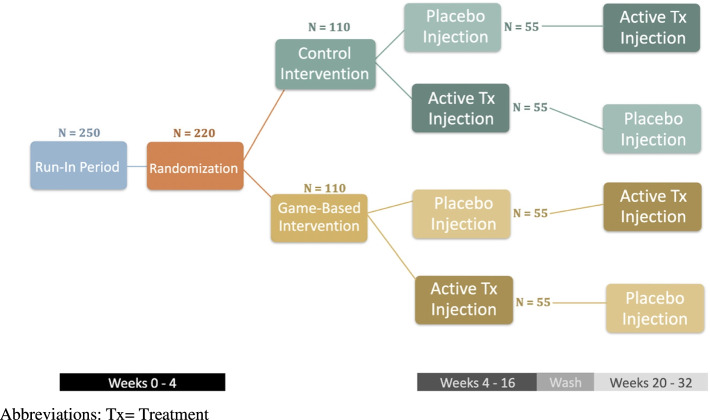
Study schema for the 32-week trial. Abbreviations: Tx, treatment

Figure 1 shows the study schema. The study protocol has been approved by the Veterans Affairs (VA) Central Internal Review Board (CIRB) (Protocol Number: 1656824–1). Initiation of recruitment is planned for March 2022.

## Methods: participants, interventions, and outcomes

### Study setting {9}

The study will recruit at four medical centers within the VA Health System across the continental United States (Omaha, Puget Sound, Washington D.C., and Philadelphia VA Medical Centers). Participants can be identified in multiple ways including screening of local clinics, query of electronic medical record data, and through referrals from local clinicians. Participants may receive “opt-out” letters before receiving phone calls from the research team. Participants who voice interest and who answer appropriately to several pre-screen questions may be invited to participant in an initial visit where consent will be obtained.

### Eligibility criteria {10}

The *inclusion criteria* include:

1. Clinical diagnosis of chronic KOA from a treating provider.
2. Age between 40 and 85 years.
3. Kellgren-Lawrence (KL) X-ray grade ≥ 1.
4. The treating physician believes joint injection is indicated or the patient has previously received injections for palliation.

The *exclusion criteria* include:

1. Contraindication to receipt of joint injection (i.e., intra-articular hardware).
2. Lack of a smartphone allowing interaction with the WTH platform.
3. Presentation for acute exacerbation of KOA.
4. Regular use of an assistive device for ambulation or inability to walk 2 blocks.
5. Poorly controlled crystalline arthritis or other diagnosis of inflammatory arthritis.
6. Treating provider or lead-site investigator (LSI) believes life expectancy is less than 1 year.
7. Based on LSI or treating provider discretion, illnesses felt to limit participant’s ability to participate in exercise.
8. Non-English speakers or poor reading ability limiting the ability to interact with text messages.

The eligibility criteria are aimed at identifying a study population that is generalizable to those that might use these interventions in real world practice. Exclusions, however, are made to ensure that those included are safe to participate in exercise and to receive joint injections.

### Who will take informed consent? {26a}

Trained study staff that have been approved by the Institutional Review Board (IRB) will obtain consent from potential trial participants. Study staff will schedule initial study visits with potential participants at the corresponding VA medical center to review the IRB-approved consent form, answer questions, and obtain written informed consent. The participants consent to storage of research specimens for 10 years for future related research studies. Participants are also consented for future contact.

Consent visits will take place at dates and times that are most convenient for potential study participants. All participants will receive their signed copy of the consent form which can be found in the supplementary materials, as described in Section {32}.

### Additional consent provisions for collection and use of participant data and biological specimens {26b}

No additional consent provisions are needed for data collection and biological specimens.

### Interventions

#### Explanation for the choice of comparators {6b}

For the exercise incentive, the comparator arm will be an attention control. Participants in the control arm will be provided with weekly communications to remind them of their personal step goals. Additionally, messages will remind participants to continue to sync their activity monitors and to complete bi-weekly surveys. Patients in both arms will receive the same weekly text messages and bi-weekly step readjustment surveys to ensure study engagement and sufficient data acquisition. For the corticosteroid injection, the comparator arm will be a lidocaine-only injection.

#### Intervention description {11a}

##### Social incentive + gamification

Half of the participants will be randomized to participate in a game-based intervention in which they can advance through levels based on the achievement of personal step goals. These goals are selected by the participant after an observation of the average daily steps taken during the run-in period.

Seventy points will be replenished at the start of each week in order to leverage the “fresh start effect” [21]. This will be done with the intention of preventing participants from feeling that they have fallen hopelessly behind. Points will be endowed, rather than given after goal achievement, to leverage loss aversion—a concept from prospect theory that reveals that individuals are more motivated by losses than gains [22, 23]. Participants will receive “medals” (i.e., gold, silver, bronze) based on their weekly point totals to provide feedback and motivate achievement of higher levels of activity. Daily messages will be sent out to either encourage participants to continue meeting their daily step goals or to inform participants that they did not meet their step goals and have lost ten points. Every 2 weeks, participants will be given the opportunity to adjust their daily step goals if they consistently failed or surpassed their goals.

In addition to the game-based intervention, participants randomized to the social incentive + gamification arm will identify support persons (i.e., family members, friends) who will provide social support during the study period. These support persons will receive texts or emails throughout the study that specify the number of days per week in which the participants met their goals and the corresponding “medals” earned. The support persons will be encouraged to cheer on the participants and encourage their progress. If a participant is unable to identify a support person, the research coordinator will serve in that role.

##### Corticosteroid injections

The research coordinators will obtain opacified syringes from the research pharmacists and remain blinded to treatment allocation. The active treatment injections will contain 40 mg methylprednisolone acetate and 2–3 mL of 1% lidocaine. The placebo injections will contain 2–3 mL of 1% lidocaine.

Both types of injections will be provided by the participants’ normal providers at routine clinical visits. All symptomatic knees are eligible for injection and the technique for providing the injections is at the preference of the provider. If a synovial effusion is present, aspiration of the effusion will be completed prior to administration of the active or placebo injection. Participants will be made aware that they may receive either an active injection or placebo injection at either time-point and that they will not be informed of the injection type until the study is completed. The injections will be administered after all other study procedures are completed during the visits. Participants will cross-over to receive both corticosteroids and lidocaine only in random order, providing an opportunity to quantify within-person effects.

#### Criteria for discontinuing or modifying allocated interventions {11b}

Participants may choose to end participation in the study at any time and for any reason. The investigator may choose to discontinue allocated interventions if patients do not adhere to the study protocol (i.e., too many data points are missing, the activity monitor is never synced) or if related adverse events occur. Additionally, participants will be required to withdraw at the time of any surgical intervention on the knee.

#### Strategies to improve adherence to interventions {11c}

Members of the central study team will regularly monitor adherence to the protocol across all participating sites. They will maintain regular communication with each local investigator regarding study progression.

Each participant, regardless of treatment assignment, will be compensated according to their participation with the study (i.e., completion of surveys and syncing of activity monitor). Participants will receive one hundred and sixty dollars ($160) per 4-month period. Participants will lose ten dollars ($10) each week if they forget to complete their questionnaires or lose fifteen dollars ($15) each month if they have more than 1 day of data missing from their activity monitors. Additionally, the WTH platform will facilitate text messages and/or emails to remind participants to wear their activity monitors, complete their questionnaires, and expect upcoming compensation. Alerts will be sent to study staff via WTH reminding them to contact participants who are not completing study events.

#### Relevant concomitant care permitted or prohibited during the trial {11d}

Participants are allowed to continue their routine care and there is no limitation to concomitant care permitted, with the exception of intra-articular viscosupplementation during the trial. The 40 mg methylprednisolone acetate dose in the active injection allows for the provision of a single “rescue” injection of an additional 40 mg methylprednisolone acetate, if necessary, for all participants without the risk of unblinding and/or exposing them to doses that exceed 80 mg within three months. Injections with viscosupplements are not permitted during study participation.

#### Provisions for post-trial care {30}

Study participants will return to their usual care or be referred to clinical providers at the conclusion of the study.

### Outcomes {12}

#### Primary outcomes

Step counts will be measured daily through linkage of activity monitors (Fitbit™) to the WTH platform. In the primary analysis, days with step counts < 1000 steps per day will be considered missing as previously described in the *BE FIT* trial [24]. Daily steps will be averaged over the valid days of wear for the week. We will assess the percent change from the baseline run-in period for average steps per day (calculated weekly) over 32 weeks. Additionally, the change in KOOS score from baseline will be calculated via platform-administered questionnaires every 2 weeks. KOOS scoring guidelines are published and the minimum clinically important difference in improvement well established [25, 26]. In the context of KOA, evidence exists to support a direct relationship between greater daily step counts and decreased risks of mortality, heart disease, and cancer as well as reduced symptomatic burden of hip and KOA [10, 11, 27, 28]. This study will test whether an incentivized intervention geared to motivating patient activity, and/or intra-articular corticosteroid injections, can improve step counts in KOA patients.

#### Secondary outcomes

We will assess the change in Patient-Reported Outcomes Measurement Information System (PROMIS) Pain Intensity score from baseline via platform-administered survey every 4 weeks over 32 weeks. Furthermore, we will determine the change in PROMIS Fatigue from baseline via platform-administered survey. PROMIS scoring guidelines, development, and uses have been published [29–31].

Other exploratory outcomes assessed monthly will include the likelihood of achieving personal daily step goals; the likelihood of achieving a daily step count greater than 6000 daily steps [32]; the change from baseline in individual KOOS domains—pain, physical function, symptoms, ability to participate in sports and recreation, and quality of life [25]; the change in PROMIS Pain Behaviors, General Life Satisfaction, and Pain Interference; the change in pain catastrophizing; the change in use of opioid medications; and the change in Pittsburgh Sleep Quality Index (PSQI). Please see the scoring guidelines for KOOS domains, PROMIS, pain catastrophizing scale, and PSQI [25, 29–31, 33, 34].

Additionally, the study team will store synovial fluid specimens from study injection visits for exploratory analyses.

### Participant timeline {13}

Table 2 provides an overview of the participant timeline. Participation will last approximately 8 months, including three research visits at the study site. Participants will use the WTH platform to set their step goals and to receive daily reminders to sync their activity monitors. The study team will administer study questionnaires via the WTH platform during the baseline visit and in 2-week and 4-week follow-up intervals.

**Table 2:** Schedule of enrollment, intervention, and assessments

		**Study period**		
	**Enrollment**	**Intervention period 1**	**Washout**	**Intervention period 2**
**Timepoint**	**Weeks 0–4**	**Weeks 4–16**	**Weeks 16–20**	**Weeks 20–32**
**Enrollment:**				
Eligibility screen	X			
Kellgren-Lawrence	X			
Informed consent	X			
Way to Health setup	X			
Fitbit™ setup	X			
Randomization *(Week 4)*	X			
**Interventions:**				
Exercise Incentive		X		X
Injection 1		X		
Injection 2				X
**Assessments:**				
Medical history	X			
Biweekly KOOS	X	X	X	X
Monthly PROMIS		X	X	X
Monthly ICOAP		X	X	X
Monthly PSQI		X	X	X
KOFBeQ		X	X	X
IPAQ		X	X	X
Collection of synovial fluid		X		X

*Abbreviations*: *KOOS* Knee Injury and Osteoarthritis Outcome Score, *PROMIS* Patient-Reported Outcomes Measurement Information System, *ICOAP* Measure of Intermittent and Constant Osteoarthritis Pain, *PSQI* Pittsburgh Sleep Quality Index, *KOFBeQ* Knee Osteoarthritis Fears and Beliefs Questionnaire, *IPAQ* International Physical Activity Questionnaire

Before participants are fully enrolled, they will be scheduled for initial screening visits at the study sites to determine eligibility. During this screening/enrollment visit, study staff will review all related procedures, obtain informed consent, and ask participants to complete baseline questionnaires. Participants will be provided with activity monitors that are linked to the WTH platform. After completion of the initial visit, there will be a 2- to 6-week run-in period in which the participants will wear their activity monitors and sync them with the WTH platform daily. If participants do not regularly use or sync their activity monitors during this run-in period, they may be withdrawn from the study.

Following the run-in period, participants will be randomized into one of four study arms and scheduled for two knee injections. Participants randomized into either of the exercise incentive study arms will be asked to share their progress with a support partner and will receive daily motivational reminders via the WTH platform. The first injection will take place after the run-in period and the second injection will take place approximately 14 to 18 weeks post-first injection. The provider will aspirate the participant’s knee(s), provide any excess synovial fluid to the study team for exploratory biomarker analyses, and administer the injections from opacified syringes.

### Sample size {14}

#### Aim 1

In the MOVE-OK pilot study, we observed an effect size of approximately 1500 steps increase from baseline for the behaviorally enhanced gamification incentive. For the average person, 1000 steps is about half of one mile [35, 36]. A study of 220 subjects will provide > 85% power to detect an increase of 1003 in average steps per day assuming 10% dropout and *α* = 0.05.

#### Aim 2

In our preliminary study, we observed a moderate effect size on the total KOOS score, favoring corticosteroid injections (*d* = 0.52). A study of 220 patients would have 97% power to detect effect sizes of *d* = 0.52 and 80% power to detect more moderate effects consistent with prior studies (*d* = 0.40) [37], assuming a 10% dropout rate. If similar effects are observed in the crossover analysis, we can expect > 99% power in the crossover analysis even after assuming a dropout rate of 20%. Sample size calculations were performed on Stata software (v. 14.2*, StataCorp, LP, College Station, TX*).

### Recruitment {15}

Participants will be identified in multiple ways including screening of local clinics, query of electronic medical record data, and through referrals from local clinicians. The local teams will place posters and pamphlets containing study descriptions and study team contact information in clinical spaces. Patients will be able to call or email the research coordinators directly to discuss participation in the study. In addition, the central team will query medical records from each participating VA site to identify patients with a diagnosis of KOA who have been seen at each site within the past year or study staff may approach participants at their clinic visits if their provider has indicated their patient’s potential interest. The local coordinators will review these lists and pre-screen through medical records to identify any exclusions. Potential participants will receive “opt out” letters and receive follow-up phone calls approximately 1 week later to determine interest in participation.

## Assignment of interventions: allocation

### Sequence generation {16a}

The WTH platform software randomizes participants and assigns them to one of the intervention arms during the randomization visit. Participants can be randomized into to one of 4 groups based on the factorial design. Several stratification variables are utilized including site, gender, and baseline step count.

### Concealment mechanism {16b}

To digitally conceal the injection type, the WTH platform codes the intervention arms ‘A’ or ‘B’ and all pharmacy orders contain non-explicit wording. To conceal the physical appearance of the intervention, syringes are opaque with the contents covered by a label and placed in a sealed bag.

### Implementation {16c}

Research staff will enroll study participants and assist them with the creation of WTH accounts. At randomization, the WTH platform will generate the allocation sequence and assign interventions. The research pharmacists will have access to WTH to view the participants’ assigned interventions and will maintain a key to the blinded naming (i.e., which intervention is ‘A’ and ‘B’).

## Assignment of interventions: blinding

### Who will be blinded {17a}

For the duration of the study, all trial participants, coordinating research staff, and investigators will be blinded to the type of injection received. The only unblinded staff will be the research pharmacists that will prepare the intervention injections, but they will not interact directly with the participants. The biostatistician involved in data analysis will also be blinded to the intervention.

## Procedure for unblinding if needed {17b}

In the unlikely scenario where unblinding is necessary, a formal request will be placed to the research pharmacist from the local site investigator who will share the information with relevant clinical staff and the participant.

## Data collection and management

### Plans for assessment and collection of outcomes {18a}

The change in steps from baseline will be assessed over all time-points (32 weeks) through linkage of the WTH platform to activity monitors. All survey links will be sent directly to participants on their mobile devices every 2 weeks irrespective of intervention arm. As described, the KOOS is a validated and widely used instrument to assess the impact of knee osteoarthritis on pain, function, and quality of life [25, 26]. Additional measures will be collected through WTH on either a bi-weekly or monthly basis and include (Table 2) PROMIS, PSQI, Intermittent and Constant Osteoarthritis Pain (ICOAP), Knee Osteoarthritis Fears and Beliefs Questionnaire (KOFBeQ), and the International Physical Activity Questionnaire (IPAQ). For more information on validation and scoring, please consult the respective sources [29–31, 33, 34, 38–40].

Given the functionality of the WTH platform, participants will have a week to complete surveys before the link expires, be prompted to complete missed questions prior to survey submission, and receive platform and staff reminders to ensure accuracy and completion of surveys. Staff will be able to contact participants immediately in order to edit erroneous or missing data in real time.

### Plans to promote participant retention and complete follow-up {18b}

The WTH platform not only tracks all study events and their completion but also sends messages and reminders to both participants and study staff. Participants will receive unique links for each study event and multiple reminders to complete events during their respective windows. Participants are also compensated based on the number of events they complete, and the WTH platform reminds them of the compensation that is lost if an event is missed. Additionally, research staff will receive daily reminders to contact participants if expected study events have not yet been completed. As a supplementary strategy to improve study retention and adherence, participants will be given promotional materials such as pins, pens, and tote bags during the randomization visit. All approaches to improve retention will be irrespective of the treatment arm.

If participants withdraw from the study, discontinue participation, or otherwise deviate from the protocol, their status can be changed on the WTH platform so that there is no further data collection. Data already collected may be used depending on its validity and completion.

### Data management {19}

Primary data capture will occur through the WTH platform. The platform coding generates staff alerts for missing, erroneous, or incomplete data entry. All staff members have access to a shared drive behind the VA firewall with standardized operating procedure templates, study visit checklists, instructional videos, and local tracking files to ensure high quality of data collection, to track (S)AEs and payment, and to minimize the risk of protocol deviations and other errors. The lead site will offer training sessions in addition to biweekly team meetings for research staff to raise questions or concerns.

### Confidentiality {27}

The WTH platform is HIPAA compliant and is the primary instrument for data collection. All data will be stored according to unique, random, patient identifiers generated for the purposes of the study. This data will be located on the secure/firewalled servers for the University of Pennsylvania Biomedical Informatics Consortium (BMIC) Data Center in data files that are protected by multiple password layers. These data servers are maintained in a guarded facility behind several locked doors, with very limited physical access rights. They are also cyber-protected by extensive firewalls and multiple layers of communication encryption. For more information, please visit the WTH Legal & Privacy page [41].

Local data will be stored on VA-approved and protected research servers at each individual institution behind the VA firewall. Paper records will be kept in locked filing cabinets in electronically secured buildings. After the conclusion of study procedures, all datasets will be cleaned of personally identifiable information before they are exported for analysis. The likelihood of loss of confidentiality is very low given the information security and privacy requirements that are in place.

### Plans for collection, laboratory evaluation, and storage of biological specimens for genetic or molecular analysis in this trial/future use {33}

At the time of injection visits, if excess synovial fluid from joint aspirations is available, the treating physician will notify research staff and collect the fluid in tubes containing sodium heparin or lithium heparin. All excess synovial fluid will be labeled with de-identified information and stored at the coordinating site in Co-Investigator’s secure, laboratory for future molecular studies. These samples will be used for studies related to the proposed research and covered by the existing IRB, destruction when no additional studies are planned. No genetic analysis is proposed in this trial. Participants consent for future use of data and biospecimens as well as recontact (Section {26a}), though additional IRB approvals would be needed if not related to the current study protocol.

## Statistical methods

### Statistical methods for primary and secondary outcomes {20a}

For Aim 1, a comparison of baseline characteristics across the exercise intervention arms will be evaluated to ensure that randomization resulted in balance of covariables. A CONSORT diagram will be generated and the retention for each study arm will be assessed. The number of missing data-points for primary and secondary outcomes will be quantified over the study follow-up and evaluated by the treatment arm. The primary analyses will use an intention-to-treat approach but secondary analyses will explore consistency with a per-protocol approach. The primary outcome of change in steps from baseline will be assessed over all time-points (over 8 months) using linear mixed-effect models with participants as a random effect. Mixed effect models, which allow for varying follow-up time and missing weekly measurements, give us flexibility using all of the available data. These models will test effects of the exercise incentive on the overall change in weekly average step counts from baseline over the entire follow-up period. Multiplicative interaction terms will be included in regression models to test for interactions between the exercise incentive and the receipt of corticosteroid injections.

For Aim 2, a comparison of baseline characteristics across the injection intervention arms will be evaluated. The primary analysis will use a linear mixed-effect model to test effects of corticosteroid injections on the change in KOOS score over all observations across treatment groups over three months. Linear mixed effect models will test treatment effects on the overall change in steps counts during each 3-month period adjusting for average values prior to the injection. A time and treatment interaction approach will be added to the mixed effect model to assess the trend of change within the follow-up period.

Secondary outcomes, including achievement of a minimal clinically important improvement, change in average daily step counts, PROMIS scores, pain catastrophizing, PSQI, KOOS domains, and use of opioid medications, will be assessed using a similar longitudinal modeling approach.

### Interim analyses {21b}

There will be no interim efficacy analyses or stopping rules.

### Methods for additional analyses (i.e., subgroup analyses) {20b}

Since all participants will receive both types of injection, the study design provides an opportunity to perform a crossover analysis. The value is the added statistical power and complete balance of patient-level factors. However, a limitation of the study design is the inherent risk of carryover or sequence effects. One treatment may seem more favorable due to the sequence in which it was received. Initial analyses will describe the participants that completed both injections and are therefore eligible for crossover analyses. All time-points within 12 weeks of the injections will be included in these crossover analyses. The first-order carryover effect will be assessed by testing for intervention-by-period interactions.

There will also be several planned stratified analyses including by biologic sex, those with higher KL grade, those with low baseline step counts, and those with low KOOS scores at baseline.

### Methods in analysis to handle protocol non-adherence and any statistical methods to handle missing data {20c}

Several measures will be taken to assess the robustness of the primary analysis. Pattern-mixed models will be used to assess the potential influence of missing data. Subjects will be divided into groups depending on their missing-data patterns and the variables based on their missing-data pattern will be used as covariates. Sensitivity analyses will also assess the impact of missing data by evaluating a Last Observation Carried Forward (LOCF) approach.

### Plans to give access to the full protocol, participant-level data, and statistical code {31c}

Upon reasonable request, the study team may be permitted to share the protocol, patient-level data, and statistical code with approval of the VA Office of Research and Development.

## Oversight and monitoring

### Composition of the coordinating center and trial steering committee {5d}

The coordinating center is at the Corporal Michael J. Crescenz VA Medical Center and includes the PI, co-PI, a program manager, and a team of research coordinators. While there is no formal steering committee, the core investigative team includes experts in rehabilitation, exercise interventions, rheumatology, clinical trials, and biostatistics. There is also a Data Safety Monitoring Committee (DSMC), which is discussed below.

### Composition of the data monitoring committee, its role, and reporting structure {21a}

A DSMC has been established and includes 3 experts in conduct of clinical trials, epidemiology/biostatistics, and physical activity interventions. They will meet annually to discuss recruitment, adverse events, and any other unexpected deviations or events. This committee is independent from study sponsors. Its members will disclose any potential conflicts of interest.

### Adverse event reporting and harms {22}

On a bi-weekly basis, participants will be asked to report any visits to an emergency room, hospitalizations, or flares of joint pain on the WTH platform. Research staff will contact participants who report these events and determine if they are related to the trial procedures. In addition, research staff will flag participants’ electronic health records and receive alerts for hospitalizations occurring at their local sites. Research staff will follow IRB guidelines when reporting events to the central and local IRB. Given the nature of the adverse events reported in the pilot study, increased hospitalizations due to comorbidities (i.e., diabetes, hyperlipidemia, chronic kidney disease, etc.) are unrelated to study procedures and to be expected. Any serious, related, or unexpected adverse event will be reported to the IRB and reported in trial publications. The study will not use any standardized coding system for adverse events.

### Frequency and plans for auditing trial conduct {23}

The principal investigator is responsible for auditing trial conduct and reporting to the IRB and DSMC. The local research office will perform periodic review of consents at each site and the central and local IRB may request a study audit at any time.

### Plans for communicating important protocol amendments to relevant parties (i.e., trial participants, ethical committees) {25}

Protocol amendments are submitted to the VA Central IRB for approval and disseminated to local sites through bi-weekly meetings. Participants will be notified if changes to the protocol modify the risk/benefit profile of the study.

### Dissemination plans {31a}

Results of the trial will be shared through publication in peer-reviewed journals. Data access may be provided on reasonable request.

## Discussion

Osteoarthritis is an extremely common and burdensome health condition with relatively few effective therapies. The MOVE-OK trial will use a pragmatic approach to understanding whether incentive-based gamification can improve physical activity (i.e., step counts) in patients with KOA and whether corticosteroids injections reduce pain and disability in this patient population.

We plan to fill current gaps in the literature using an innovative factorial trial design that efficiently addresses both research questions within a single randomized trial. In addition, the crossover element of our design lowers participant variation and improves statistical power by allowing us to observe the effect of both injection treatments (corticosteroid vs. lidocaine) on each participant. The use of remote wearable technology is innovative and will measure primary outcomes in real-world settings. This allows for the collection of accurate data on participants’ pain and disability while reducing both the intrusiveness of the measurement and potential clinical biases. The highly virtual nature of data collection is also a strength in that it may improve retention and limit interruptions in data capture. Challenges in scheduling in-person visits are reduced, for example, due to individual or systemic barriers (i.e., pandemic restrictions).

We also propose to evaluate the effects of these interventions in particular subgroups including biologic sex, those with more advanced radiographic disease, those with low baseline step counts, and those with low KOOS scores at baseline. In addition, exploratory analyses will evaluate the impact of pain catastrophizing, centralized pain, sleep quality, and the use of opioid and non-opioid analgesics. Exploratory analyses will also evaluate synovial fluid characteristics to better understand how inflammatory changes in the joint may predict response to different interventions, potentially leading to new predictive biomarkers. These analyses will help identify the types of patients that are most likely to respond to these interventions and help to inform more precise guidelines in this area.

This trial has some limitations worth noting. Using a factorial design can increase the complexity of data analysis and participant randomization, as well as the possibility of interactions between the two treatments. As each participant receives both treatments after the crossover, there is a potential for order effects: the first injection received (i.e., Corticosteroids) may have an effect on the efficacy of the second injection received (i.e., lidocaine) or vice-versa. We also chose a 40 mg dose for the trial in order to allow for unblinded rescue injections; however, higher doses may be necessary to achieve a more meaningful effect. Further, missing data is a common source of bias in clinical trials. In this case, a clear approach is needed for managing missing step counts and survey data. A plan to replace participants that withdraw can be considered in order to keep the treatment comparisons balanced. Lastly, the use of a smartphone and the WTH platform presents a technological and economic barrier that might not make the study completely generalizable to the veteran population as whole. The study team will intervene if participants encounter technological issues to help maintain participation. Implementation of these interventional approaches might benefit from a focus on improving access to the technology.

In summary, the findings of this trial will provide insight into the treatment of KOA in two important ways. The study will determine if incentive-based gamification can improve physical activity within a KOA population in order to reduce pain and improve function. The study will also quantify the efficacy of corticosteroids by providing outcomes measured at frequent time points in real-life conditions, including outcomes related to pain AND physical activity. Results will help influence future guidelines and health care policy surrounding these interventions.

## Trial status

The current protocol version is 2 (9/13/2021). Recruitment is expected to begin March 2022 and will end approximately March 2025.

## Supplementary Information

**Additional file 1.**

## Data Availability

Datasets may be available through a formal request process through the principal investigator and co-investigators. Data will not be shared without the approval of CIRB and Philadelphia VA IRB. When shared, data will be de-identified and anonymized.

## Abbreviations

BMIC: Biomedical Informatics Consortium
CIRB: Central Institutional Review Board
DSMC: Data Safety Monitoring Committee
IRB: Institutional Review Board
ICOAP: Intermittent and Constant Osteoarthritis Pain
IPAQ: International Physical Activity Questionnaire
K-L: Kellgren-Lawrence
KOA: Knee osteoarthritis
KOFBeQ: Knee Osteoarthritis Fears and Beliefs Questionnaire
KOOS: Knee Osteoarthritis Outcome Score
LOCF: Last Observation Carried Forward
MOVE-OK: Marching On for VEterans with Osteoarthritis of the Knee
PSQI: Pittsburgh Sleep Quality Index
PROMIS: Patient-Reported Outcome Measurement Information System
VA: Veterans Affairs
WTH: Way to Health

## References

[1] Golightly YM, Allen KD, Jordan JM (2016). Defining the burden of osteoarthritis in population-based surveys. Arthritis Care Res (Hoboken).

[2] Rivera JC, Amuan ME, Morris RM, Johnson AE, Pugh MJ (2017). Arthritis, comorbidities, and care utilization in veterans of operations enduring and Iraqi Freedom. J Orthop Res.

[3] Deshpande BR, Katz JN, Solomon DH, Yelin EH, Hunter DJ, Messier SP, Suter LG, Losina E (2016). Number of persons with symptomatic knee osteoarthritis in the US: impact of race and ethnicity, age, sex, and obesity. Arthritis Care Res (Hoboken).

[4] Balsamo S, Diniz LR, dos Santos-Neto LL, da Mota LM (2014). Exercise and fatigue in rheumatoid arthritis. Isr Med Assoc J.

[5] Kelley GA, Kelley KS, Hootman JM (2015). Effects of exercise on depression in adults with arthritis: a systematic review with meta-analysis of randomized controlled trials. Arthritis Res Ther.

[6] Knittle KP, De Gucht V, Hurkmans EJ, Vlieland TP, Peeters AJ, Ronday HK, Maes S (2011). Effect of self-efficacy and physical activity goal achievement on arthritis pain and quality of life in patients with rheumatoid arthritis. Arthritis Care Res.

[7] Iversen MD, Brawerman M, Iversen CN (2012). Recommendations and the state of the evidence for physical activity interventions for adults with rheumatoid arthritis: 2007 to present. Int J Clin Rheumatol.

[8] Rongen-van Dartel SA, Repping-Wuts H, Flendrie M, Bleijenberg G, Metsios GS, van den Hout WB, van den Ende CH, Neuberger G, Reid A, van Riel PL, Fransen J (2015). Effect of aerobic exercise training on fatigue in rheumatoid arthritis: a meta-analysis. Arthritis Care Res.

[9] Larkin L, Kennedy N (2014). Correlates of physical activity in adults with rheumatoid arthritis: a systematic review. J Phys Act Health.

[10] Wellsandt E, Golightly Y (2018). Exercise in the management of knee and hip osteoarthritis. Curr Opin Rheumatol.

[11] Kolasinski SL, Neogi T, Hochberg MC, Oatis C, Guyatt G, Block J, Callahan L, Copenhaver C, Dodge C, Felson D, Gellar K, Harvey WF, Hawker G, Herzig E, Kwoh CK, Nelson AE, Samuels J, Scanzello C, White D, Wise B, Altman RD, DiRenzo D, Fontanarosa J, Giradi G, Ishimori M, Misra D, Shah AA, Shmagel AK, Thoma LM, Turgunbaev M, Turner AS, Reston J (2020). 2019 American College of Rheumatology/Arthritis Foundation Guideline for the Management of Osteoarthritis of the Hand, Hip, and Knee. Arthritis Rheumatol.

[12] Khoja SS, Almeida GJ, Freburger JK (2020). Recommendation rates for physical therapy, lifestyle counseling, and pain medications for managing knee osteoarthritis in ambulatory care settings: a cross-sectional analysis of the National Ambulatory Care Survey (2007–2015). Arthritis Care Res (Hoboken).

[13] Kahneman D (2011). Thinking fast and slow.

[14] Thaler RH, Sunstein CR (2008). Nudge: improving decisions about health, wealth, and happiness.

[15] O'Donoghue T, Rabin M (2000). The economics of immediate gratification. J Behav Decis.

[16] Chapman GB, Coups EJ (2006). Emotions and preventive health behavior: worry, regret, and influenza vaccination. Health Psychol.

[17] Jeffery RW, Gerber WM, Rosenthal BS, Lindquist RA (1983). Monetary contracts in weight control: effectiveness of group and individual contracts of varying size. J Consult Clin Psychol.

[18] Juni P, Hari R, Rutjes AW, Fischer R, Silletta MG, Reichenbach S, da Costa BR. Intra-articular corticosteroid for knee osteoarthritis. Cochrane database Syst Rev. 2015(10):CD005328. Epub 2015/10/23. doi: 10.1002/14651858.CD005328.pub3. PubMed PMID: 26490760. 10.1002/14651858.CD005328.pub3 PMC8884338 26490760

[19] Khan M, Bhandari M (2018). Cochrane in CORR®: intra-articular corticosteroid for knee osteoarthritis. Clin Orthop Relat Res.

[20] McAlindon TE, LaValley MP, Harvey WF, Price LL, Driban JB, Zhang M, Ward RJ (2017). Effect of intra-articular triamcinolone vs saline on knee cartilage volume and pain in patients with knee osteoarthritis: a randomized clinical trial. JAMA..

[21] Dai H, Milkman KL, Riis J (2014). The fresh start effect: temporal landmarks motivate aspirational behavior. Manage Sci.

[22] Kahneman D, Tversky A (1979). Prospect theory: an analysis of decision under risk. Econometrica.

[23] Patel MS, Asch DA, Rosin R, Small DS, Bellamy SL, Heuer J, Sproat S, Hyson C, Haff N, Lee SM, Wesby L, Hoffer K, Shuttleworth D, Taylor DH, Hilbert V, Zhu J, Yang L, Wang X, Volpp KG (2016). Framing financial incentives to increase physical activity among overweight and obese adults: a randomized, controlled trial. Ann Intern Med.

[24] Patel MS, Benjamin EJ, Volpp KG, Fox CS, Small DS, Massaro JM (2017). Effect of a game-based intervention designed to enhance social incentives to increase physical activity among families: the BE FIT randomized clinical trial. JAMA Intern Med.

[25] Roos EM, Roos HP, Lohmander LS, Ekdahl C, Beynnon BD (1998). Knee Injury and Osteoarthritis Outcome Score (KOOS)–development of a self-administered outcome measure. J Orthop Sports Phys Ther.

[26] Roos EM, Toksvig-Larsen S (2003). Knee injury and Osteoarthritis Outcome Score (KOOS) - validation and comparison to the WOMAC in total knee replacement. Health Qual Life Outcomes.

[27] Conigliaro P, Triggianese P, Ippolito F (2014). Insights on the role of physical activity in patients with rheumatoid arthritis. Drug Dev Res.

[28] van VeldhuijzenZanten JJ, Rouse PC, Hale ED (2015). Perceived barriers, facilitators and benefits for regular physical activity and exercise in patients with rheumatoid arthritis: a review of the literature. Sports Med (Auckland, NZ).

[29] Lee AC, Driban JB, Price LL, Harvey WF, Rodday AM, Wang C. Responsiveness and minimally important differences for four Patient-Reported Outcomes Measurement Information System (PROMIS) short forms: physical function, pain interference, depression, and anxiety in knee osteoarthritis. J Pain. 2017. https://www.ncbi.nlm.nih.gov/pubmed/28501708 10.1016/j.jpain.2017.05.001 PMC5581239 28501708

[30] Lai JS, Cella D, Choi SW, Junghaenel DU, Christodoulou C, Gershon R, Stone A (2011). How item banks and their application can influence measurement practice in rehabilitation medicine: a PROMIS fatigue item bank example. Arch Phys Med Rehabil.

[31] Gershon R, Cella D, Rothrock N, Hanrahan RT, Bass M (2010). The use of PROMIS and assessment center to deliver patient-reported outcome measures in clinical research. J Appl Meas.

[32] White DK, Tudor-Locke C, Zhang Y, et al. Daily walking and the risk of incident functional limitation in knee osteoarthritis: an observational study. Arthritis Care & Research. 2014;66(9):1328-1336. 10.1002/acr.22362. 10.1002/acr.22362 PMC4146701 24923633

[33] Sullivan MJL, Bishop SR, Pivik J (1995). The Pain Catastrophizing Scale: development and validation. Psychol Assess.

[34] Buysse DJ, Reynolds CF, Monk TH, Berman SR, Kupfer DJ (1989). The Pittsburgh Sleep Quality Index: a new instrument for psychiatric practice and research. Psychiatry Res.

[35] Singh JA, Luo R, Landon GC, Suarez-Almazor M (2014). Reliability and clinically important improvement thresholds for osteoarthritis pain and function scales: a multicenter study. J Rheumatol.

[36] Angst F, Aeschlimann A, Michel BA, Stucki G (2002). Minimal clinically important rehabilitation effects in patients with osteoarthritis of the lower extremities. J Rheumatol.

[37] Patel MS, Asch DA, Volpp KG (2015). Wearable devices as facilitators, not drivers, of health behavior change. JAMA.

[38] Moreton BJ, Wheeler M, Walsh DA, Lincoln NB (2012). Rasch analysis of the intermittent and constant osteoarthritis pain (ICOAP) scale. Osteoarthritis Cartilage.

[39] Benhamou M, Baron G, Dalichampt M (2013). Development and validation of a questionnaire assessing fears and beliefs of patients with knee osteoarthritis: the Knee Osteoarthritis Fears and Beliefs Questionnaire (KOFBeQ). PLoS One.

[40] Hagströmer M, Oja P, Sjöström M (2006). The International Physical Activity Questionnaire (IPAQ): a study of concurrent and construct validity. Public Health Nutr.

[41] Balachandran, M. (n.d.). Penn Way to Health. Retrieved March 20, 2022, from https://www.waytohealth.org/legal/

