# Supplementary material for: Protocol for a multi-center randomized controlled trial to evaluate the benefits of exercise incentives and corticosteroid injections in osteoarthritis of the knee (MOVE-OK)
Source: Trials. 2022 Jul 27;23:604. doi: 10.1186/s13063-022-06529-w (PMC9327347; doi:10.1186/s13063-022-06529-w)
Supplement: Supplementary file 1 — Additional file 1. [file 13063_2022_6529_MOESM1_ESM.pdf]

Department of Veterans Affairs

RESEARCH CONSENT FORM

***Version Date:** 10/18/2021*

Participant Name: ____ Date:__________

Title of Study: A Pragmatic Trial to Determine the Benefit of Behaviorally Enhanced Exercise Incentives and Corticosteroid Injections in Osteoarthritis of the Knee: Marching On For VEterans with Osteoarthritis of the Knee ("MOVE-OK")

Principal Investigator: Joshua Baker VA Facility: Corporal Michael J. Crescenz VA Medical Center

Principal Investigator for Multisite Study: Joshua Baker

KEY SUMMARY INFORMATION ABOUT THIS STUDY

You are being invited to take part in a research study that is being funded by VA Rehabilitation Research and Development. Before you decide to take part, it is important for you to know why the research is being done and what it will involve. This includes any potential risks to you, as well as any potential benefits you might receive. Taking part in this study is completely voluntary.

WHAT IS THE STUDY ABOUT AND HOW LONG WILL IT LAST?

If you participate in this study, you will be given a Fitbit activity monitor and you will sign up to the study website and download the Fitbit app. The website will have your phone number and you will receive text messages from our team. You may be asked to choose a support person to root you on. During the study you will receive two injections. One only contains numbing medicine and one also contains corticosteroids (depomedrol). The study will enroll about 40 veterans.

By doing this study, we hope to learn how to help veterans increase their physical activity and reduce symptoms of arthritis. Your participation in this research will last about 8-10 months.

Another purpose of this research is to gather information on the safety and effectiveness of joint injections with depomedrol (a steroid like "Cortisone"), an FDA-approved treatment for knee osteoarthritis that you have been getting or were planning to get. The use in the study is consistent with labeling indications.

WHAT ARE KEY REASONS YOU MIGHT CHOOSE TO VOLUNTEER FOR THIS STUDY?

You may be interested in volunteering if you have longstanding knee pain. You may also consider participating if you are interested in increasing your physical activity. For a complete description of benefits, refer to the "Detailed Information" section of this document.

WHAT ARE KEY REASONS YOU MIGHT CHOOSE NOT TO VOLUNTEER FOR THIS STUDY?

If you participate, you will not receive your usual injections and you will receive an injection at some point that does not include corticosteroids ("Cortisone") but only numbing medicine. You will have to provide your cell phone number to the website and sign up to the Fitbit app. For a complete description of risks, refer to the Research Details section of this document. *If* you do not want to participate, you can continue your usual care with your doctor. For a complete

FOR VA CENTRAL IRB USE ONLY

PI/SC Approval Date: **09/14/21**

LSI Approval Date: **01/10/21**

LSI Verification Date: **N/A**

description of alternate treatment/procedures, refer to the Research Details section of this document.

DO YOU HAVE TO TAKE PART IN THE STUDY?

If you decide to take part in the study, it should be because you really want to volunteer. You will not lose any services, benefits or rights you would normally have if you choose not to volunteer.

WHAT IF YOU HAVE QUESTIONS, SUGGESTIONS OR CONCERNS?

The person in charge of the study is Dr. Joshua Baker at the Corporal Michael J. Crescenz VA Medical Center.] If you have questions, suggestions, or concerns regarding this study or you want to withdraw from the study his/her contact information is: [redacted]

DETAILED INFORMATION ABOUT THE STUDY

WHAT IS THE PURPOSE OF THIS STUDY?

The purpose of this research study is to see if corticosteroid (cortisone) injections and web-based and other encouragements to increase physical activity can promote physical activity and improve disability and pain in patients with knee osteoarthritis. With this research we hope to learn whether interacting with a website and receiving text messages and other feedback from the study and/or other family members or friends can help veterans increase their physical activity and, in doing so, reduce the symptoms of their arthritis. Another purpose of this research is to see if steroid injections are safe and effective for treating your knee osteoarthritis. This treatment has been approved by the Food and Drug Administration for use in treating this problem. Patients may be eligible to participate if they are able to walk a half mile and have received or have been recommended by a clinical provider to receive steroid injections for osteoarthritis of the knee. So you may be eligible to participate even if you have never received steroid injections before.

HOW LONG WILL I BE IN THE STUDY?

This research study is expected to take approximately 3 years to complete. Your individual participation will be approximately 10 months. You will have a total of 3 research visits at the Corporal Michael J. Crescenz VA Medical Center in Philadelphia. Each visit will take about 2 hours. We aim to recruit 220 total participants throughout the country with approximately 40-60 at each individual VA site.

FOR VA CENTRAL IRB USE ONLY

PI/SC Approval Date: 09/14/21

LSI Approval Date: 01/10/21

LSI Verification Date: N/A

WHAT WILL HAPPEN IF I TAKE PART IN THE STUDY?

Before you are fully enrolled in the study, you will be scheduled for an initial visit at the Corporal Michael J. Crescenz VA Medical Center to see if this is the right study for you to participate in. This visit will take place in the Rheumatology Clinic or in the [redacted]

During this visit, your informed consent for participation will be obtained, study staff will review all study related procedures, and ask you to fill out questionnaires about your feelings of your knee pain and other questions regarding your medical history. You will be provided with a Fitbit activity tracker and asked to download the Fitbit application on your smartphone and register on the Way to Health platform to link your Fitbit account. Study staff will assist you with setting these things up. You will have to sign up to the Fitbit application and enter your name and email address. You will also be prompted by the Fitbit application to enter your date of birth so that the program can calculate your age. However, if you do not feel comfortable sharing your date of birth you do not have to. You will not be required to pay for any devices or applications.

You then will be shown how to use the Way to Health mobile platform, which is a software application developed at the University of Pennsylvania. This application allows the ability to connect with your Fitbit activity tracker, which will remotely send the study team your daily activity levels. You will also be asked to use this platform to set your steps goals and fill out the required study questionnaires at various time points within the study (more information is provided below). This initial visit will take approximately 2 hours.

After completion of your initial visit, there is a 2-6 week trial period to see if this is the right study for you to participate in. During this time, you will be expected to wear your Fitbit activity tracker each day and "sync" it with the Way to Health platform. Syncing means to merge the data from the Fitbit to the Fitbit App on the smartphone. Usually this is automatic. If you do not sync your Fitbit regularly or use the Fitbit regularly during this time, you might be asked not to continue in the study.

Following this period, you will be scheduled for two knee injections. While you may have received injections as part of your usual clinical care, the injections in this study will be different as explained below. The first of these injections will occur after your next research visit and the second will occur about 4 months after your first injection. If you currently receive injections every 3 months, you may have to wait an extra month between the injections. These injections

will take place within the Rheumatology Clinic located on the 1st floor of the CMCVAMC in Module E. Each injection visit will take approximately two hours.

Prior to your first injection visit, you will meet with study staff for another research visit. Based upon your average steps measured by the Fitbit device, you will be asked to choose a step goal that you would like to reach. You will receive a daily reminder to sync your Fitbit. Depending on each individual's cell phone plans, text messaging fees may apply.

You will be asked to complete a series of questionnaires using the Way to Health website about your medical history, pain medication use, pain, pain related behaviors, feelings about your knee pain, physical activity, disability, fatigue and sleep quality. These surveys will be asked at different times in follow-up as well. You will answer questionnaires at your initial visit, prior to both injection visits, and every 2 weeks during your study participation. These surveys are expected to take between 10 and 25 minutes to complete each time you take them. You are free to skip any questions that you would prefer not to answer.

The study coordinator will also use an "algometer" to measure your sensitivity to pain at each injection visit (2 separate times). The algometer is pressed on different areas of your body. You will be asked to tell the coordinator performing the test when you start to experience pain. This can cause some minor pain similar to what you might experience during a physical exam with your doctor.

The next part of the study involves two different "randomization" steps. Randomization is like a flip of a coin. One randomization will be the type of injection you get. The other randomization will be to determine what incentives you get to exercise. You will be placed into one of 4 different study arms.

At your injection visits, you will meet with the study coordinator or local site investigator and have an injection administered by a physician or nurse practitioner. If you have a clinical provider that has already provided your injections, they may be able to continue to give the injections. However, you might receive your injection from another provider that is part of the study team. You will have a chance of receiving one of two injections. Some injections will include both corticosteroids (cortisone) and lidocaine (numbing medicine), while others will receive only lidocaine (numbing medicine) injections. This assignment is completely random and you will not know which one you will be receiving until you have completed your study participation. The syringe will have tape on it so that you cannot see what is being injected. Therefore, one of the two injections you get during your participation in this study will **not** be

FOR VA CENTRAL IRB USE ONLY

PI/SC Approval Date: 09/14/21

LSI Approval Date: 01/10/21

LSI Verification Date: N/A

with cortisone, and this is different than what you would be getting if you did not participate in this study.

If you develop pain after an injection, we will help you get an urgent appointment in the rheumatology clinic. If you and your provider believe you would benefit from another injection, you will be permitted to receive another injection at this visit. Being in the study means that there is a chance you would receive an additional injection which carries the same risk as the other injections.

The other randomization that occurs in this study is whether you will have social incentives or not. If you are assigned to a group with incentives, you will receive incentives to exercise and be asked to share your progress with a family member or friend. You will be asked to enter their telephone number into the website so they can receive information about your exercise. You may also receive a report of your step performance via text message or email. We will also send you a text message to remind you to sync the Fitbit with your phone and to complete assessments every 2 weeks.

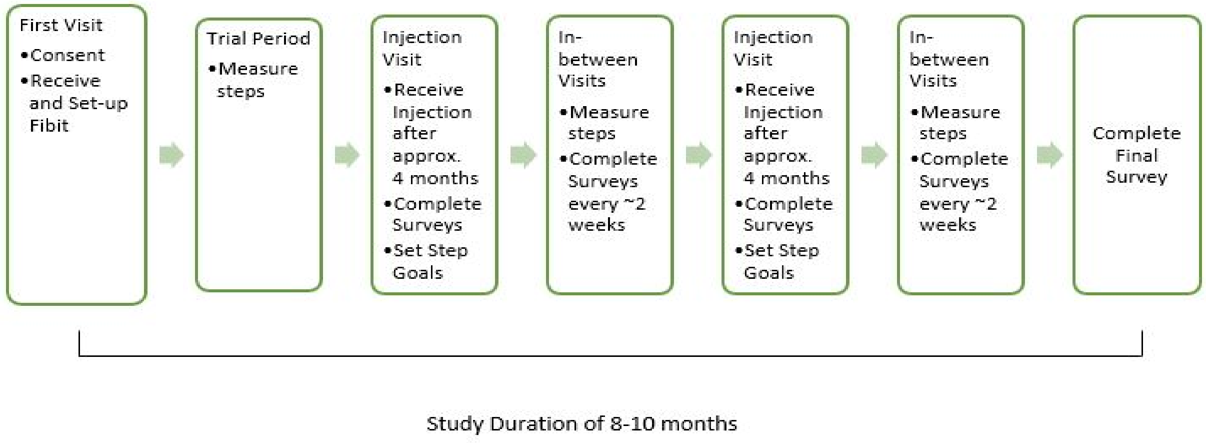

The image above is an overview of the visitation schedule (described previously) if you consent to participate in the study.

FOR VA CENTRAL IRB USE ONLY

PI/SC Approval Date: 09/14/21

LSI Approval Date: 01/10/21

LSI Verification Date: N/A

During the course of your participation in this study, we will be leaving research notes in your chart. In these notes, we will include clinically relevant information related to your participation in this study. Study results such as your survey responses and step counts will not be shared with your clinical providers. This will include whether you received an injection, though the medication you received will not be put in the notes.

All study procedures will be done at the Corporal Michael J. Crescenz VA Medical Center in Philadelphia.
If your clinical provider collects synovial fluid from your joint when you receive the injection, we would like to store and use any leftover fluid to test for factors in your synovial fluid that might be associated with characteristics of your disease or to help predict who is most likely to respond to treatment. This fluid will be sent to the Corporal Michael J. Crescenz VA Medical Center in Philadelphia and labeled with your study identifier. You will not be able to profit from any commercial product developed from your specimens.

WHAT IS EXPECTED OF ME IF I TAKE PART IN THIS STUDY?

- Attend all three study visits and receive both injections as part of your participation.
- Keep your study appointments. If you miss an appointment, please contact the investigator or research staff to reschedule as soon as you know you will miss the appointment.
- Fill out your surveys and sync your Fitbit at least every month as instructed.
- Ask questions as you think of them.
- Tell the investigator or research staff if you believe you might be pregnant, or are planning to become pregnant.
- While participating in this research study, do not take part in any other research project without approval from the investigators. This is to protect you from possible injury from things such as extra blood drawing, extra X-rays, or potential drug interactions. Taking part in other research studies without first discussing it with the investigators of this study may invalidate the results of this study, as well as that of the other studies.

WHAT POSSIBLE RISKS OR DISCOMFORTS MIGHT I HAVE IF I TAKE PART IN THIS STUDY?

Risks associated with injection: If you are being enrolled in this study, you have been receiving cortisone injections or have planned to receive them. The doctor or nurse practitioner performing the procedure will discuss the risks of the procedure before performing the procedure and you will sign a separate consent for the procedure. These injections can be

FOR VA CENTRAL IRB USE ONLY

PI/SC Approval Date: 09/14/21

LSI Approval Date: 01/10/21

LSI Verification Date: N/A

associated with pain and swelling where the needle enters the skin. Local bruising is also possible. Approximately 1 in 10,000 to 50,000 people receiving an injection will get a joint infection which can require hospitalization, surgery, and/or treatment with antibiotics. If you are on anticoagulant blood thinners you are at a higher risk in this study of bleeding into the joint. A possible risk is that you may not receive as much benefit in terms of pain with the lidocaine only injection. If, as a result, you require another injection to reduce your pain, the same risks of an injection will apply to that extra procedure. You will also receive a standard dose of the steroid if you participate in the study. If you have been receiving a different dose up until now, you will be required to get a different dose that may affect how well it works.

Risks associated with Algometer: You may feel some tenderness in the areas where the pressure is being measured.

Risks associated with Fitbit: You may feel uncomfortable wearing the Fitbit for extended periods of time. You may experience minor frustration with syncing the Fitbit to the Way to Health platform. If you have any questions, please feel free to contact the study staff at any time. Finally, increasing your physical activity could possibly result in injury or a flare of your arthritis.

Risks from sharing your information: Your directly identifiable information will be recorded by the study team and stored on the VA servers in a password-protected folder. Your other study information (step counts and survey results) will be stored on the Way to Health platform at the University of Pennsylvania along with a study identifier (a unique code). Your name and date of birth will not be stored on the University of Pennsylvania database. However, you will be required to share your phone number and/or e-mail address with the Way to Health platform so that you can receive text messages and/or emails. The Way to Health platform is protected with software that is similar to what is used to protect medical record information.

You may also feel uncomfortable about sharing your activity progress with social contacts you may designate if randomized to the group that are getting incentives. You might also experience anxiety about receiving updates on your progress by text message and/or emails every day.

There is always a chance that any procedure can harm you. The procedures in this study are no different. In addition to the risks described above, you may experience a previously unknown risk or side effect.

Risks of usual care you receive are not risks of this study. Those risks are not included in this consent form. You should talk with your health care providers if you have any questions about the risks of usual care.

FOR VA CENTRAL IRB USE ONLY

PI/SC Approval Date: 09/14/21

LSI Approval Date: 01/10/21

LSI Verification Date: N/A

WHAT ARE THE POSSIBLE BENEFITS OF THIS STUDY?

You may not benefit from participating in this research study. The study might benefit you by resulting in an increase in your physical activity level and reducing the symptoms of your arthritis. Additionally, this study may lead to information that can aid in the clinical care of people with osteoarthritis of the knee.

WHAT OTHER CHOICES DO I HAVE IF I DO NOT WANT TO JOIN THIS STUDY?

You have the choice not to participate in this research study. Participation is voluntary and you do not have to participate if you do not want to. You may choose not to participate and continue with the routine care (and get injections) provided by your primary rheumatologist.

HOW WILL MY PRIVATE INFORMATION BE PROTECTED?

During this study, we will collect personal information such as:

- Name,
- Address,
- Telephone number,
- Email address,
- Date of birth,
- Social security/Medical record number,
- Personal medical history,
- Results from physical examinations,
- Tests or procedures, and nicotine and alcohol use.

Your name and social security/medical record number will be used only as necessary by the study staff at at the Corporal Michael J. Crescenz VA Medical Center in Philadelphia. The funding agency will not have access to your study data. You will be required to enter your name and phone number into the web-based "Way To Health" platform at the University of Pennsylvania. Participants will also be required to enter their support partners name and phone number. The "Way To Health" platform will collect your step information from the FitBit app but will not collect information such as your date of birth, or address. Participants will create a Fitbit account that will be linked to WTH, but no personal health information (PHI) will be exchanged through this. Fitbit will ask for a name and email address but participants may use false names.

FOR VA CENTRAL IRB USE ONLY

PI/SC Approval Date: 09/14/21

LSI Approval Date: 01/10/21

LSI Verification Date: N/A

To protect the confidentiality of your records, all written or printed documents collected for this research study will be stored in a locked file cabinet in a locked office at the Corporal Michael J. Crescenz VA Medical Center in Philadelphia. Any electronic research data collected will be stored on a secure sever behind the VA firewall that will only be accessible by study staff. As explained previously, because some study activities will be done using the Way to Health platform some personal information (cell phone number and/or email address), will be stored within the system.

If you have an accident or reaction during the course of the study, your entire medical record may be used and disclosed as clinically necessary.

Internal monitors from the Central Institutional Review Board (IRB), a research oversight committee, may inspect study records for quality assurance.

This informed consent document will be added to your medical record. We will include information about your study participation in your medical record.

The results of this study may be published; however, you will not be identified by name or other personal identifiers. Further, your medical records will not be revealed unless required or authorized by law.

All research records, including the investigator's research records, must be retained according to the National Archives and Records Administration VHA's Records Control Schedule.

A description of this clinical trial will be available on http://www.ClinicalTrials.gov as required by U.S. Law. This website will not include information that can identify you. At most, the website will include a summary of the results. You can search this website at any time.

Health Information Portability and Accountability Act (HIPAA)

There are rules to protect your private health information. Federal and state laws and the federal medical law, known as the HIPAA Privacy Rule, also protect your privacy. By signing this form, you provide your permission called your 'authorization,' for the use and disclosure of information protected by the HIPAA Privacy Rule.

The research team working on the study will collect information about you. This includes things learned from the procedures described in this consent form. They may also collect other information including your name, address, date of birth, and information from your medical

records such as your medical history. You will be required to enter your name and phone number into the web-based "Way To Health" platform at the University of Pennsylvania. The "Way To Health" platform will also collect your step information from the Fitbit app and the answers you provide to surveys throughout the study, but will not collect any information such as your date of birth, or address.

The research team may also need to disclose your health information and the information it collects to others as part of the study progress. Others may include the Central Institutional Review Board, the local Institutional Review Board, Office of Human Research Protections (OHRP), the VA Office of Research Oversight (ORO), and the Government Accountability Office (GAO). Your health information disclosed pursuant to this authorization may no longer be protected by Federal laws or regulations and may be subject to re-disclosure by the recipient.

While this study is being conducted you will not have access to your research related health records. This will **not** affect your VA healthcare, including your doctor's ability to see your records as part of your normal care and will **not** affect your right to have access to the research records after the study is completed.

You can revoke this authorization, in writing, at any time. To revoke your authorization, you must write to the Release of Information Office at this facility or you can ask a member of the research team to give you a form to revoke the authorization. Your request will be valid when the Release of Information Office receives it. If you revoke this authorization, you will not be able to continue to participate in the study. This will not affect your rights as a VHA patient to treatment or benefit outside of the study.

If you revoke this authorization, Dr. Joshua Baker and the research team can continue to use information about you that was collected before receipt of the revocation. The research team will not collect information about you after you revoke the authorization.

Treatment, payment, or enrollment/eligibility for benefits cannot be conditioned on you signing this authorization. This authorization will expire at the end of the research study unless revoked prior to that time.

For biospecimens that are collected as part of this study, identifiers will be removed from your biospecimens and, after such removal, the information or biospecimens could be used for research related to the study and could be distributed to another investigator to perform research tests without additional informed consent from you.

WHAT ARE THE COSTS TO ME IF I TAKE PART IN THIS STUDY?

You will not be charged for any treatments or procedures that are part of this study. If you usually pay co-payments for VA care and medications, you will still pay these co-payments for VA care and medications that are not part of this study.

WILL I BE COMPENSATED FOR MY TIME?

You will be compensated for your participation in the study. The amount you will receive will be based upon the study activities that you actually complete (detailed below). You will be sent a check or voucher for the amount earned approximately every 3 months. You will also be able to keep your Fitbit at the end of the study. Checks will be sent from Corporal Michael J. Crescenz VA Medical Center in Philadelphia.

For your participation, you will be compensated:

- $30 for your initial visit
- $30 for each injection visit
- $30 for completing the end of study questionnaires.

You will also receive:

- $10 for each set of surveys completed (occurring every 2 weeks, 14 surveys total)
- $15 each month for syncing the Fitbit at least once a month.

Total participation equals up to $410 over the course of 10 months if you complete all tasks. The amount you receive will be based upon the study activities you actually complete. You will be sent a check for each amount about every 3 months. You will also be able to keep your Fitbit at the end of the study.

WHAT WILL HAPPEN IF I AM INJURED BECAUSE OF MY BEING IN THE STUDY?

If you are injured as a result of taking part in this study, the VA will provide necessary medical treatment at no cost to you unless the injury is due to non-compliance by a study participant with study procedures or if the research is conducted for VA under contract with an individual or non-VA institution.

FOR VA CENTRAL IRB USE ONLY

PI/SC Approval Date: **09/14/21**

LSI Approval Date: **01/10/21**

LSI Verification Date: **N/A**

Compensation is not available should an injury occur. You do not give up any legal rights or release the VA from any liability by signing this form.

If you should have a medical concern or get hurt or sick as a result of taking part in this study, call:

DURING THE DAY:

Dr. [redacted]

AFTER HOURS:

The [redacted]

DO I HAVE TO TAKE PART IN THE STUDY?

You understand that you do not have to take part in this study, and your refusal to participate will involve no penalty or loss of rights to which you are entitled. You may withdraw from this study at any time without penalty or loss of VA or other benefits to which you are entitled. If you withdraw, you may be asked to return for a final study visit in order to assure your safety. You should withdraw in writing using the Revocation of Authorization for Use & Release of Individually Identifiable Health Information for Veterans Health Administration Research form. Even if you withdraw, we can continue to use information about you that has been collected up to that point, and specimens already collected cannot be withdrawn and can be used for research purposes. No information will be collected after you formally withdraw in writing.

This study is expected to end after all participants have completed all visits, and all information has been collected. Physical therapy is also available as a treatment.

RIGHT OF INVESTIGATOR TO TERMINATE MY PARTICIPATION

This study may also be stopped at any time without your consent because:

- The Principal Investigator feels it is necessary for your health or safety. Such an action would not require your consent, but you will be informed if such a decision is made and the reason for this decision.
- You have not followed study instructions. For example, not syncing your Fitbit or not completing surveys for a prolonged period.

FOR VA CENTRAL IRB USE ONLY

PI/SC Approval Date: 09/14/21

LSI Approval Date: 01/10/21

LSI Verification Date: N/A

- You repeatedly lose your Fitbit.
- Your medical condition changes or if you require frequent hospitalizations.
- The Sponsor or the Principal Investigator has decided to stop the study.

If your study participation is stopped early, you will keep the Fitbit and continue your medications as directed by your primary care physician. The study team will help ensure you have follow-up with your usual arthritis doctor. Early withdrawal from the study poses no health risks for you.

WHO DO I CONTACT ABOUT THIS STUDY IF I HAVE QUESTIONS?

In case there are medical problems, research related injuries or questions, you have been told that you should call Dr. Joshua Baker at [redacted] or the study coordinator at [redacted] during the day. After hours, you should call the VA operator at [redacted] and ask for [redacted]. If the operator is unable to reach Dr. Joshua Baker, you should ask to speak to the rheumatology provider on call.

If you have questions about your rights as a study participant, or you want to make sure this is a valid VA study, you may contact the VA Central Institutional Review Board (IRB). This is the Board that is responsible for overseeing the safety of human participants in this study. You may call the VA Central IRB toll free at [redacted] if you have questions, complaints or concerns about the study or if you would like to obtain information or offer input.

WILL I BE TOLD NEW INFORMATION ABOUT THIS STUDY?

Sometimes during the course of a research study, new information becomes available about the treatment that is being studied that might change a person's decision to stay in the study. If this happens, your research doctor will tell you about it and discuss with you whether you want to continue in the study. If you decide to withdraw from the study, your research doctor will arrange for your medical care to continue. If you decide to continue in the study, you might be asked to sign an updated informed consent form. Your research doctor could also decide it to be in your best interests to withdraw you from the study. If so, he or she will explain the reasons and arrange for your usual medical care to continue. You should not expect that any clinically-relevant research results will be provided to you from this study.

WHO COULD PROFIT FROM THE STUDY RESULTS?

Biospecimens collected as part of this study will not be used for profit.

FOR VA CENTRAL IRB USE ONLY

PI/SC Approval Date: 09/14/21

LSI Approval Date: 01/10/21

LSI Verification Date: N/A

DOES THIS STUDY INVOLVE GENETIC RESEARCH AND HOW WILL MY GENETIC INFORMATION BE PROTECTED?
This study does not involve any genetic research.

RE-CONTACT
Sometimes additional research questions become of interest after the completion of a research study. You might be re-contacted by the study investigators to ask you if you would like to participate in future studies. You are not obligated to participate in any future studies.

TISSUE COLLECTION AND STORAGE
Synovial fluid specimens will be collected and sent to Dr. Cala Scanzello’s laboratory at the Corporal Michael J. Crescenz VA Medical Center in Philadelphia ([redacted]). Only Dr. Scanzello, her research team, and principal investigator, and the co-investigators will have access to specimens. Specimens will be retained 10 years before they will be destroyed.

AGREEMENT TO PARTICIPATE IN THE RESEARCH STUDY

Dr./Mr./Ms______________________________ ______________________________________has explained the research study to you. You have been told of the risks or discomforts and possible benefits of the study. You have been told of other choices of treatment available to you. You have been given the chance to ask questions and obtain answers.

By signing this document below, you voluntarily consent to participate in this study and authorize the use and disclosure of your health information in this study. You also confirm that you have read this consent, or it has been read to you. You will receive a copy of this consent after you sign it. A copy of this signed consent will also be put in your medical record.

**I agree to participate in this research study as has been explained in this document.**		
____________________ Participant’s Name	____________________ Participant’s Signature	__________ Date

FOR VA CENTRAL IRB USE ONLY

PI/SC Approval Date: **09/14/21**

LSI Approval Date: **01/10/21**

LSI Verification Date: **N/A**
